# Takotsubo Syndrome (TTS) in Onco-Hematologic Patients: Retrospective Analysis and Focus on the Correlation or Not With Anticancer Drugs. Case Reports and Review of the Literature

**DOI:** 10.3389/fonc.2022.875391

**Published:** 2022-05-26

**Authors:** Manlio Monti, Pietro Cortesi, Roberto Vespignani, Ilaria Bronico, Chiara Gallio, Michele Flospergher, Laura Matteucci, Giovanni Luca Frassineti

**Affiliations:** ^1^ Department of Medical Oncology, Istituto di Ricovero e Cura a Carattere Scientifico (IRCSS) Istituto Romagnolo per lo Studio dei Tumori (IRST) “Dino Amadori”, Meldola, Italy; ^2^ Cardio-Oncology Unit, Istituto di Ricovero e Cura a Carattere Scientifico (IRCCS) Istituto Romagnolo per lo Studio dei Tumori (IRST) “Dino Amadori”, Meldola, Italy; ^3^ Information Technology (IT) Unit, Istituto di Ricovero e Cura a Carattere Scientifico (IRCCS) Istituto Romagnolo per lo Studio dei Tumori (IRST) “Dino Amadori”, Meldola, Italy; ^4^ Radiology Unit, IRCCS Istituto Romagnolo per lo Studio dei Tumori (IRST) “Dino Amadori”, Meldola, Italy

**Keywords:** Takotsubo syndrome, monoclonal antibody, cancer, cardiovascular toxicity, adverse drug reactions, case report

## Abstract

Takotsubo syndrome (TTS) is an uncommon cardiovascular condition also known as stress-induced cardiomyopathy or broken heart disease. The syndrome, characterized by acute non-coronary segmental ventricular dysfunction, commonly occurs as a reaction to severe emotional or physical stress and can cause significant problems. Several classes of chemotherapeutic agents that are known to be cardiotoxic have been shown to be associated with TTS in cancer patients. Describing a case of TTS from chemotherapy and/or monoclonal antibody is important because these drugs are widely used and their temporary or permanent suspension could compromise the success of treatment. The detection and reporting of suspected adverse drug reactions in clinical practice are the foundations of postmarketing surveillance. We performed a retrospective analysis of a large number of patients followed at our cancer centre to identify drugs that could lead to the onset of TTS, focusing our attention on 2 monoclonal antibodies, bevacizumab and rituximab plus chemotherapy. A search was carried out for the word “Takotsubo” in database sources such as in PubMed, in medical oncology, radiology and cardiology electronic clinical records. From October 2007 to March 2021, of the 79,005 patients seen or treated for any kind of malignancy at our institute, 9 had a diagnosis of TTS (4 before and 5 after the diagnosis of malignancy). Only 2 patients had TTS after treatment with the anticancer drugs, bevacizumab and rituximab plus chemotherapy. These two patients were hospitalised, one for subocclusion while the other for pulmonary embolism (PE) with a life threatening condition and in need of intravenous catecholamines. For both patients, an ECG, echocardiography and coronary angiography were performed as well as blood tests with a subsequent diagnosis of TTS and both received cardiological treatment with resolution of the clinical picture. A reassessment of the two cases found that a subocclusion and intravenous catecholamines appeared to be the most likely triggers. In conclusion, TTS is rare in cancer patients. Identifying TTS triggers could be useful because it could induce therapeutic changes.

## Introduction

Takotsubo cardiomyopathy was first described in 1991 by Sato et al. (1). The characteristic apical ballooning of this condition in ventriculography resembles a takotsubo, a round-bottomed pot with a narrow neck used in Japan as an octopus trap, hence the reason for its unusual name (2). Takotsubo syndrome (TTS), also known as ‘broken heart disease’, is a stress-induced cardiomyopathy that commonly occurs as a reaction to severe emotional or physical stress and can cause significant problems. Patients with TTS have symptoms that mimic acute coronary syndrome, such as shortness of breath, hypotension, and chest pain. As a diagnosis of TTS is reached by a process of elimination, an angiogram generally rules out blocked coronary arteries, whereas an echocardiogram shows left ventricular (LV) segmental dysfunction. The LV dysfunction caused by TTS usually resolves within a few weeks. Although the majority of patients fully recover within two months of diagnosis, serious complications may occur during the acute phase.

Whilst the underlying pathophysiology of TTS is not completely understood, it is believed to result from a multitude of predisposing factors and pathogenic mechanisms. The syndrome is often preceded by acute stress followed by chest pain, electrocardiographic abnormalities and elevated cardiac troponin levels without, however, the presence of obstructive coronary artery disease. Several classes of chemotherapeutic agents known to be cardiotoxic have been found to be associated with TTS in cancer patients (3). TTS begins to be considered an epiphenomenon of cardiotoxicity in patients with cancer (4). Guidelines regarding TTS management are lacking and the therapeutic strategies are based on clinical experience. Generally, in the treatment of TTS the drugs that are used are: ACE inhibitors, beta-blocker, diuretics and or nitroglycerin (if there is no left ventricular outflow tract obstruction).

In this report we evaluate the potential correlation between two monoclonal antibodies (bevacizumab and rituximab), chemotherapy and TTS; we also provide a review of the literature on the topic.

## Methods

Since 2004, our cancer institute has used electronic clinical records for all patients admitted to our Inpatient Ward or accessing the Outpatients Clinic and Day Hospital. For the present study, a search of medical oncology, radiological and cardiological records was carried out for the word “Takotsubo” to identify patients with a history of TTS. The period considered for the retrospective analysis was from 1^st^ October 2007 to 31^st^ March 2021.

During the study period, 79,005 patients were seen and/or treated at our cancer centre, and 9 were diagnosed with TTS (4 before and 5 after the diagnosis of malignancy). The comorbidities and major characteristics of these nine patients are shown in **Table 1**. The criteria used for the diagnosis of TTS have been different over the years, but we reviewed the nine cases considering new InterTAK Diagnostic Criteria (5). Only two patients developed TTS after anticancer drugs.

**Table 1:** Characteristics of patients with diagnosis of malignancies and synchronous or metachronous Takotsubo Syndrome (TTS).

Patient no.	Gender	Age^*^ (years)	Malignancy	Obesity	Hypertension	Dyslipidemia	Diabetes mellitus	Previous or current smoker	COPD	Hyper- or hypothyroidism	Anxiety or depression	History of alcohol abuse	TTS before or after cancer diagnosis	Cancer treatment before TTS	Time between last cancer treatment and TTS	In-hospital complications	Cardiac resolution
#1	F	69	Ovarian cancer	Yes	Yes	Yes	No	No	No	No	No	No	After (15 months)	Bevacizumab	15 days	LVEF <45%	Yes
#2	F	58	Lung cancer	No	No	Unk	No	Yes	No	No	No	No	Before (4 years)	–	–	n.e.	Yes
#3	F	80	Non Hodgkin’s lymphoma	No	Yes	No	No	No	No	No	No	No	After (9 months)	Rituximab	14 days	Right bundle branch block, LVEF <45%, thromboembolism	Yes
#4	F	63	Lung cancer	No	Yes	No	No	Yes	Yes	No	No	No	After (1 month)	–	–	None	Yes
#5	F	59	Glioblastoma	No	No	No	No	No	No	No	No	Unk	Before (3 years)	–	–	None	Yes
#6	F	73	Breast cancer	Yes	Yes	Yes	Yes	Yes	No	No	No	No	Before (8 months)	–	–	None	Yes
#7	F	49	Pancreatic cancer	Yes	Yes	Yes	Yes	Yes	No	Yes	No	No	After (1 month)	–	–	LVEF <45%, mitral regurgitation	Yes
#8	F	82	Colon cancer	Unk	Yes	No	No	Unk	No	No	No	No	After (1 month)	Surgery	1 month	LVEF <45%, mitral regurgitation	Yes
#9	F	90	Chronic lymphatic leukaemia	Unk	Yes	Yes	No	No	No	No	Yes	No	Before (1 year)	–	–	None	Yes

^*^Age at diagnosis of TTS.

Unk, unknown; COPD, chronic obstructive pulmonary disease; n.e., not evaluable; LVEF, left ventricular ejection fraction.

## Case 1

In the first case, we had a 69-year-old woman with peritoneal carcinomatosis from ovarian cancer. Her past medical history was remarkable for hypertension (treated with atenolol 100 mg daily), dyslipidemia, osteoporosis and cholecystectomy for gallbladder stones when she was 50 years old. She did not have any previous cardiac history. Family history was negative. She was treated with carboplatin AUC 6 in a one-hour infusion + paclitaxel 175 mg/m^2^ in a 3-hour infusion for three cycles before undergoing hysterosalpingo-oophorectomy and peritonectomy. After surgery, the patient continued the same chemotherapy, with the addition of bevacizumab 15 mg/m^2^, for another three cycles. It was then decided to continue with bevacizumab 15 mg/m^2^ in monotherapy, but 15 days after the second cycle, the patient went to her local Emergency Room because of vomiting grade 2 and dehydration with hypokalemia 3 mMol/L (3.5-5.1). The physical examination was unremarkable, blood pressure 100/70 mmHg, heart rate 82 beats per minute and she did not have chest pain. An ECG showed anterior ST segment elevation, without Q waves. Troponin was 1407 ng/L (10 ng/L). An emergency cardiac catheterization revealed a non-critical atherosclerosis and akinesia in the mid anterior and apical segments of the left ventricle wall (apical ballooning-like appearance). An echocardiography showed a non-dilated left ventricle, apical akinesis with 40% ejection fraction (EF) but no significant valve defects. Urinary metanephrine level was 791 µg (<530 µg over 24 hours).

**Figure 1:**
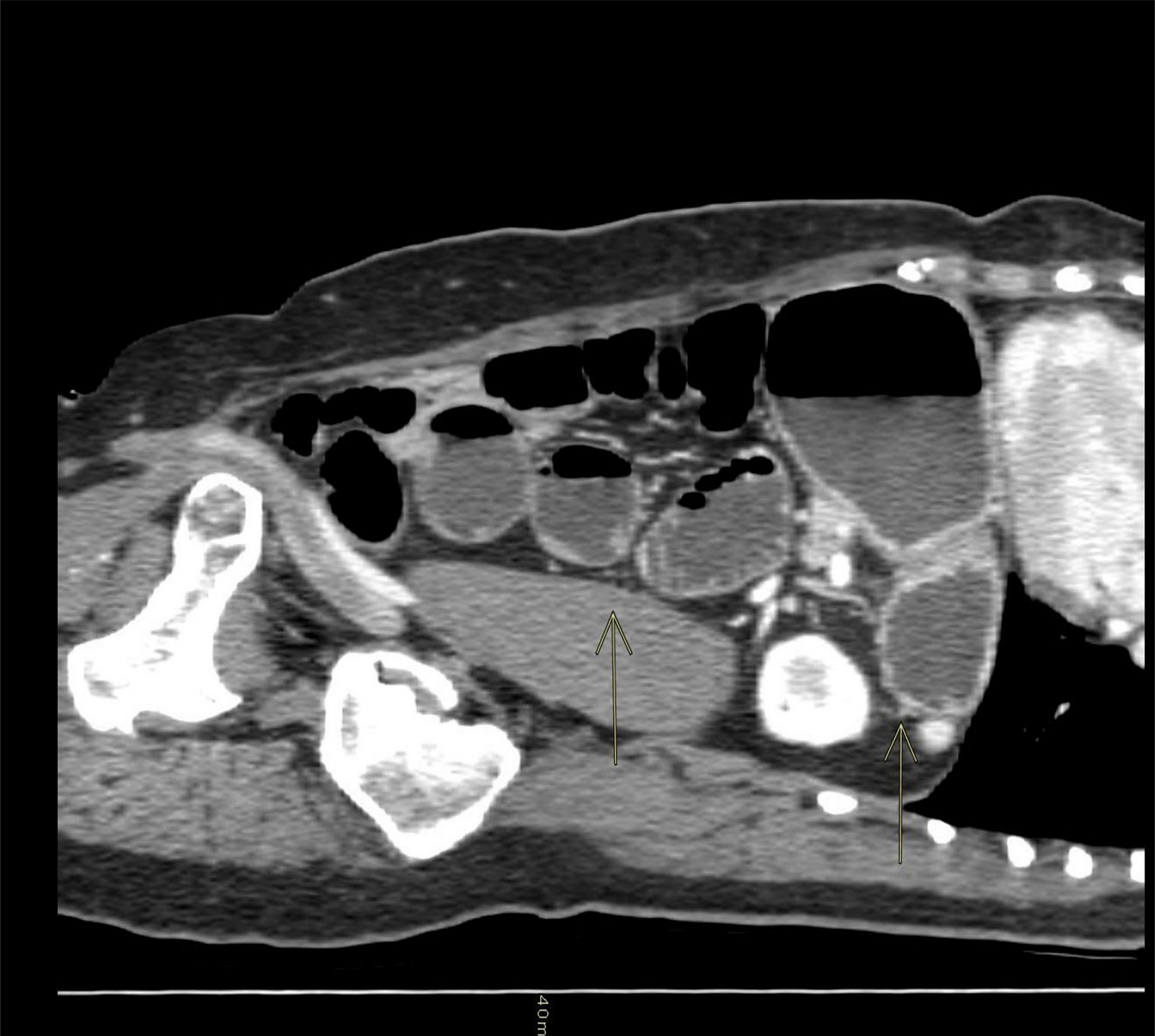
Sagittal CT scan of the abdomen: the arrows show a dilated stomach and ileal levels.

**Figure 2:**
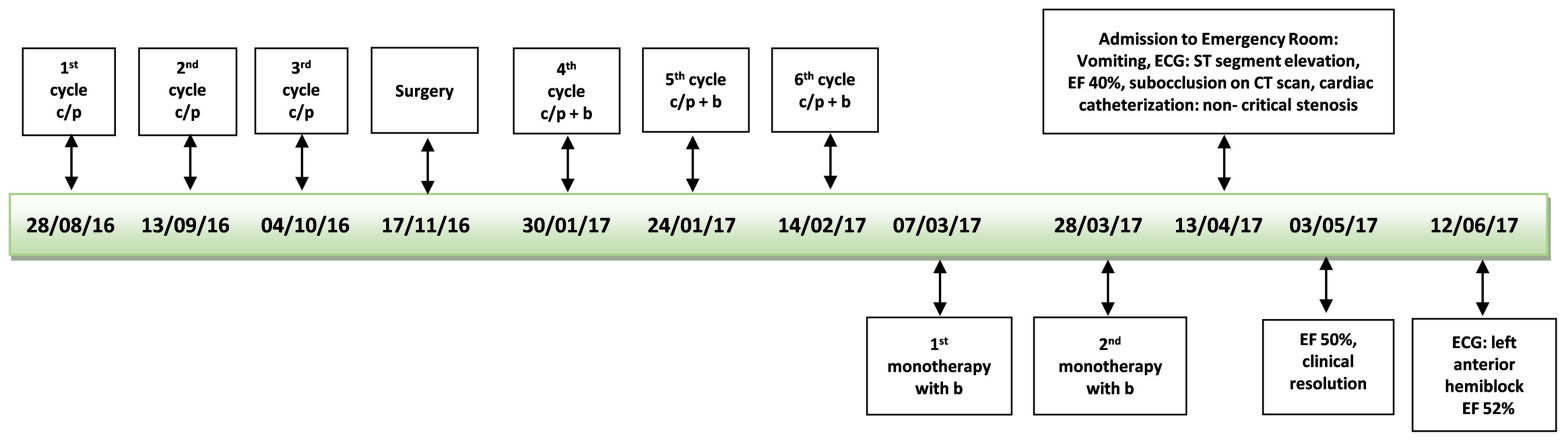
Time line first patient. *c/p*, carboplatin/paclitaxel; *b*, bevacizumab; *ECG*, electrocardiogram; *EF*, ejection fraction.

The patient continued to vomit and began to complain of abdominal pain. A brain CT scan was negative, while an abdominal CT scan revealed a dilated stomach and increased ileal levels (**Figure 1**). Arterial pressure was at the upper limit of normal (150/90 mmHg), 100 beats per minute, ambient air oxygen saturation 98%. The following treatment was administered: intravenous (i.v.) diluted potassium chloride 20 mEq daily, oral canrenoate 25 mg twice daily, oral acetylsalicylic acid 100 mg daily, oral bisoprolol 1.25 mg thrice daily, i.v. levosulpiride 25 mg twice daily, enoxaparin 4000 IU daily and, initially, i.v. furosemide 25 mg daily. Before being discharged, the patient underwent another echocardiography that showed recovery of systolic function (EF 50%) and mild apical hypokinesis. The patient was discharged with oral bisoprolol, oral canrenoate and oral acetylsalicylic acid at the same doses as in hospitalization (**Figure 2**).

Treatment with bevacizumab was immediately interrupted. Two months after the diagnosis of TTS, an ECG revealed a left anterior hemiblock, while the echocardiography showed a normal left ventricle.Two years after the suspension of bevacizumab, a PET scan confirmed disease progression and the patient underwent a different type of chemotherapy in another hospital with carboplatin AUC 5 in a 1-hour infusion and pegylated liposomal doxorubicin 30 mg/m^2^ in a 2-hour infusion the first time and a 1-hour infusion from the second cycle for six cycles and then she started niraparib 300 mg until today as maintenance therapy. She is still alive 4 years after the diagnosis of TTS.

## Case 2

In the second case, we had an 80-year-old woman with high-grade locally advanced non Hodgkin’s lymphoma of the breast. Her past medical history was remarkable for hypertension (treated with atenolol 50 mg plus chlorthalidone 12,5 mg daily) and hysterosalpingo-oophorectomy for ovarian cysts when she was 38 years old. She underwent right hemicolectomy and liver resection for metastatic colon cancer when she was 48 years old and she received six cycles of chemotherapy with oxaliplatin 85 mg/m^2^, folinic acid 200 mg/m^2^ followed by and 5 fluorouracil, as a 400 mg/m^2^ intravenous bolus then 600 mg/m^2^ infusion over 22 hours, day 1 and 2 of a 14-day cycle. She did not have any previous cardiac history. Family history was negative. In November 2018, due to lymphoma, she was treated with 6 cycles of rituximab 375 mg/m^2^, vincristine 1.4 mg/m^2^, liposomal doxorubicin 50 mg/m^2^, cyclophosphamide 750 mg/m^2^, methylprednisolone 40 mg/m^2^ day one (every three weeks). Between the fourth and fifth cycle of therapy there was an interruption of about two months because the patient asked for a suspension because of a death in the family.

Two weeks after the last cycle of chemotherapy plus rituximab, the patient presented herself at our Day Hospital with dyspnea, ambient air oxygen saturation was 80%, blood pressure 90/60 mmHg, tachyarrhythmia (115-120 beats per minute). A physical examination revealed dry skin, no edema, reduced vesicular murmur *in toto*, no lung sounds, treatable abdomen.

**Figure 3:**
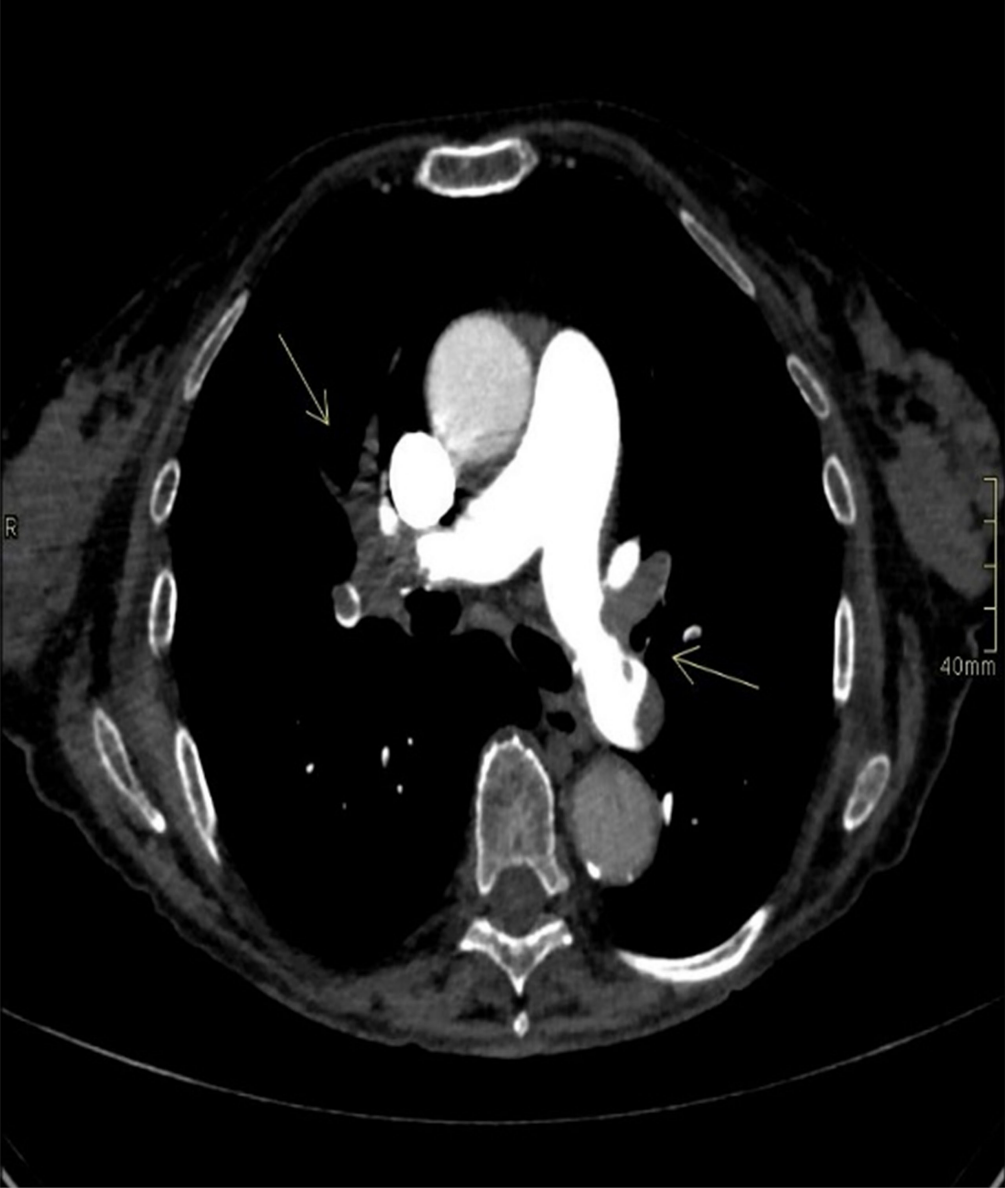
Chest CT scan: the arrows show bilateral lobar and segmental pulmonary embolism.

**Figure 4:**
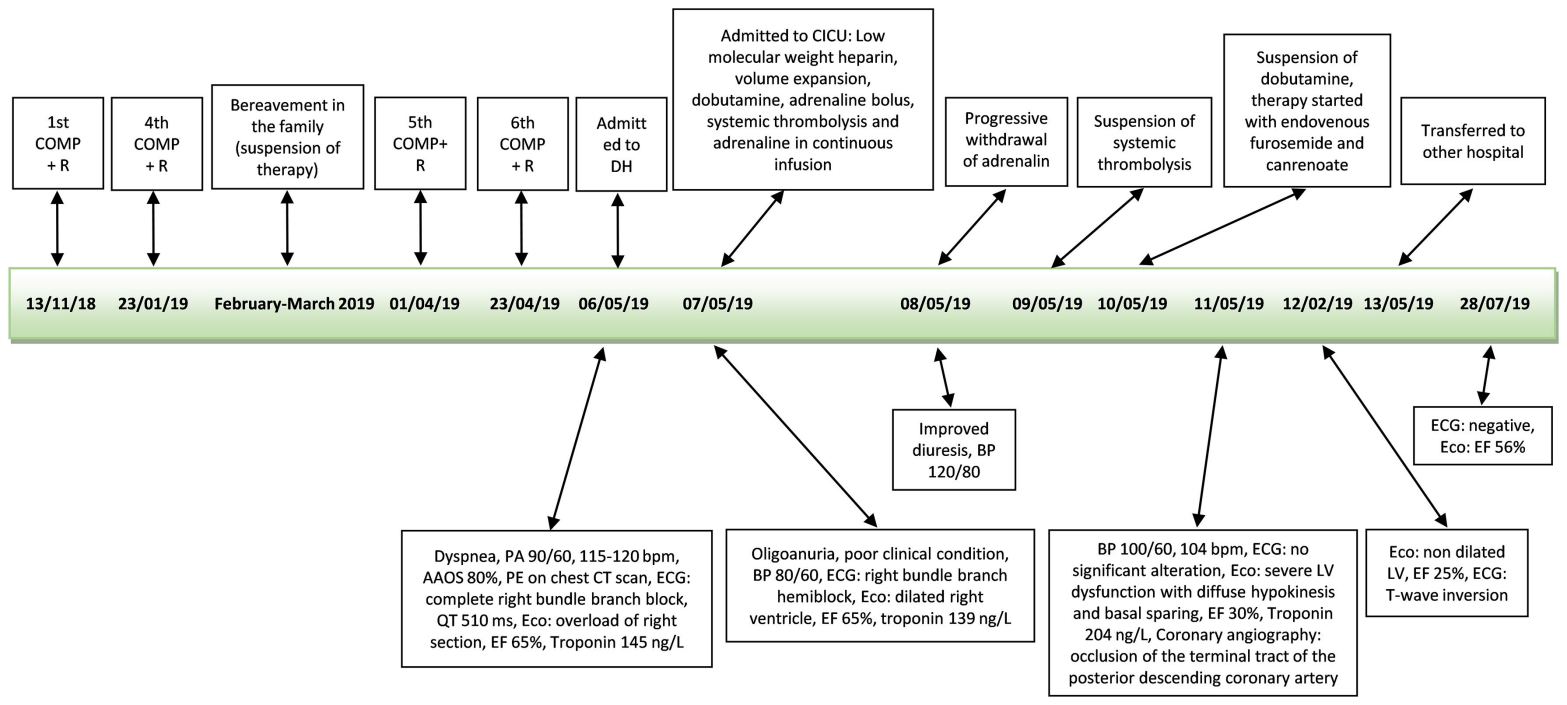
Time line second patient. *COMP+R*, vincristine, liposomal doxorubicin, cyclophosphamide+rituximab, *AAOS*, ambient air oxygen saturation; *ECG*, electrocardiogram; *bpm*, beats per minute; *EF*, ejection fraction; *BP*, blood pressure; *PE*, pulmonary embolism; *Eco*, echocardiograph; *DH*, Day Hospital; *CICU*, cardiac intensive care unit.

An ECG showed a complete right bundle branch block that had previously been absent and QT interval prolongation up to 510 ms (<460 ms). An urgent chest CT scan showed bilateral lobe and segmental pulmonary embolism (**Figure 3**). The patient underwent a venous Doppler imaging of the lower limbs that revealed a non occlusive thrombosis of the twin vein in the right leg and a thrombosis of the popliteal vein in the left leg. An echocardiography showed marked overload of the right sections and LV function with 65% EF. She was immediately admitted to the Cardiac Intensive Care Unit of the local hospital, an ECG showed a right bundle branch hemiblock and she underwent a low molecular weight heparin (6000 IU), volume expansion (physiological solution 500 mL) and inotropic therapy (dobutamine 4 mcg/kg/min). The patient’s clinical conditions worsened and she became hypotensive. The cardiologist increased dobutamine to 15 mcg/kg/min and added adrenaline bolus 0.2 mg, but without benefit. Given the patient’s critical condition, systemic thrombolysis with rt-PA (single bolus of 0.6 mg/kg equal to 30 mg) and adrenaline in continuous infusion up to 7 ml/hour were administered at the onset of anuria, with a subsequent improvement in hemodynamics and diuresis over the next few hours. A careful but rapid weaning off of inotropes followed over 24 hours and treatment proceeded with oral bisoprolol 1.25 mg daily, oral furosemide 25 mg daily and *i.v.* canrenoate 100 mg daily. However, following the onset of acute dyspnea, an echocardiogram was performed **(** **Supplementary Video S1** **)**, showing severe LV dysfunction with diffuse hypokinesis and basal sparing. The EF 30% was compatible with myocardiopathy stress and an ECG showed a T-wave inversion. A coronary angiography showed occlusion of the terminal tract of the posterior descending coronary artery. The patient’s clinical conditions rapidly improved and cardiologic therapy was modified to oral furosemide 25 mg daily, oral canrenoate 50 mg daily, oral ramipril 2.5 mg daily and oral bisoprolol 1.25 mg daily. This same therapy was maintained even at hospital discharge. Troponin dropped from 145 ng/L (10 ng/L) to 106 ng/L, NT-proBNP was 34337 ng/L (<1800 ng/L) and urinary metanephrine level was 844 µg (<500 µg over 24 hours). The patient was transferred to the hospital closer to home. Two months after the diagnosis of TTS, an echocardiogram showed EF 56% and an ECG was negative (**Figure 4**). The patient was diagnosed with a case of TTS and rituximab was suspended. She is alive and well after 2 years.

## Discussion

Several drugs, in particular, anticancer agents such as axitinib, sunitinib, bevacizumab, trastuzumab (molecular-targeted agents), some chemotherapeutic agents, and immune checkpoint inhibitors are associated with the development of TTS. There is a wide range of cardiovascular toxicities related to chemotherapy and some immunotherapies, including ischemia, vasospasm, arrhythmia, QT prolongation and acute cardiomyopathy. Chemotherapy-induced TTS is a rare but acknowledged phenomenon.

Since it was first described (1), there has been much debate as to whether or not the coronary artery is involved in Takotsubo cardiomyopathy. Coronary angiography showing LV systolic dysfunction distinguishes between TTS and myocardial infarction because TTS patients generally do not have coronary stenosis (6).

The diagnosis of TTS is based on the proposed Mayo Clinic criteria (7). However, a 2016 report by the Taskforce on TTS of the Heart Failure Association of the European Society of Cardiology proposed a new diagnostic criteria including anatomical features (transient regional wall motion abnormalities of LV, regional wall motion abnormalities, absence of culprit atherosclerotic coronary artery disease), ECG changes (ST-segment elevation, ST depression, left bundle branch block, T-wave inversion, and/or QTc prolongation) during the acute phase, cardiac biomarkers (elevated BNP or NT-proBNP and troponin during the acute phase), and reversibility of the myocardial dysfunction on cardiac imaging at follow-up (3-6 months) (8).

Currently, the literature data shows that TTS concerns 10% (range 4%-29%) of neoplastic patients (9) while in our experience the prevalence (1%) is much lower. In our opinion, the difference is potentially linked to the sample considered. In the systematic review of Pelliccia et al. (9), 19 studies were considered for a total of 1109 patients with TTS. Of these patients only 106 patients had malignancy.

In the first case describing an ST-segment elevation, a coronary angiography was performed that revealed a non-critical atherosclerosis. These elements along with the other elements collected ruled out acute coronary syndrome. For the second case that did not have an ST segment elevation, the InterTak diagnostic score can be considered. It assigns a score to a series of indicators (female sex 25 points, emotional stress 24 points, physical stress 13 points, non ST-segment depression 12 points, psychiatric disorders 11 points, neurological disorders 9 points, QT prolongation 6 points) (10). The score was 43 points (female sex, non ST segment depression, QT prolongation). A score ≤70 points correlates to a low/intermediate risk of TTS, however it cannot be excluded that the score is underestimated due to the fact that a cancer patient is likely to be more exposed to physical and emotional stress. In the low/intermediate risk the next step is to consider coronary angiography, which in this case showed an obstruction, however, the restoration of EF and ECG excluded a heart attack.

Current guidelines state that the presence of coronary artery disease does not contribute to the pathophysiologic state and transient LV dysfunction associated with TTS (5). In a study by Auzel et al. on angiographic results in TTS patients, 15.5% of cases showed evidence of coexisting coronary artery disease, suggesting that active coronary artery disease does not preclude the diagnosis of the syndrome (6). In our experience, only one of the 9 patients with TTS had coronary stenosis.

In the literature there are only 2 reports of TTS after bevacizumab (11) and one after the initial infusion of rituximab) (12). In 2015, another case of TTS was reported after pulsed methylprednisolone and rituximab (13). The patient in question had been treated with 6 cycles of fludarabine, cyclophosphamide and rituximab a few years before without any problems, and at the time of the onset of TTS was undergoing retreatment with rituximab. Kumar et al. described a case of hemophagocytic lymphohistiocytosis (HLH) in a patient with glioblastoma and anakinra and rituximab were used, which have shown to be effective in managing Epstein-Barr viremia associated HLH. In that paper the authors reported that the patient had TTS after anakinra and rituximab but they did not specify and or consider a potential correlation between these drugs and TTS (14).

In our case collection, three patients were diagnosed with TTS one month after their cancer diagnosis, which would seem to indicate a strong emotional trigger. All 9 patients with TTS were menopausal women, which may have influenced their risk of developing cardiomyopathy. In our first case the patient received three courses of carboplatin and paclitaxel before surgery and then bevacizumab was added to the same chemotherapy. In this case TTS appeared after two further courses with only bevacizumab. Franco et al. (11) described a case of TTS that appeared two days after chemotherapy (unspecified), and bevacizumab for colon cancer, while in the second case the same author described TTS that appeared three weeks after chemotherapy (unspecified) and bevacizumab in a metastatic non small-cell lung cancer. In the literature, some cases of TTS have been reported after the use of paclitaxel (15, 16) or carboplatin in combination with pemetrexed and immunotherapy (17). Actually, in our first case of TTS that appeared after bevacizumab, the symptoms that led to hospitalisation (vomiting and dehydration) are not typical symptoms of TTS. We know bevacizumab is not an emetogenic agent. The CT scan showed a dilated stomach and increased ileal levels that may be associated with carcinosis subocclusion. Various gastrointestinal symptoms such as acute cholecystitis, vomiting, and diarrhoea are considered possible triggers for TTS (8, 18). Chen-Yu et al. described a case of TTS in a patient with paralytic ileus (19) while we are reporting an uncommon case of a patient with carcinosis accompanied by TTS.

**Figure 5:**
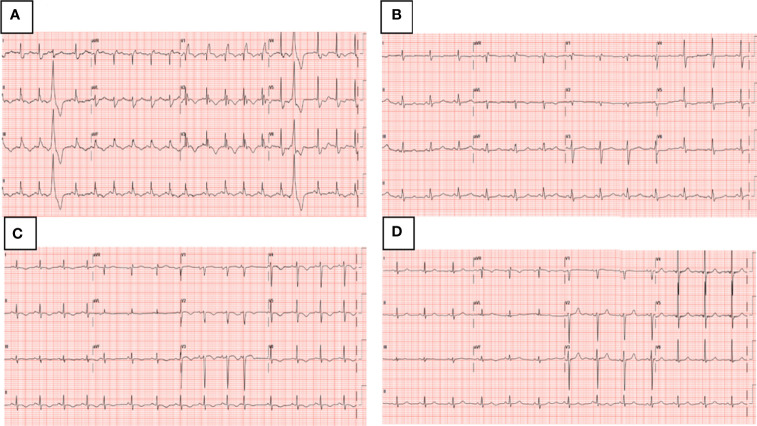
Dynamic ECG changes **(A)** ECG on admission to Day Hospital, complete right bundle branch block that has previously been absent and QT interval prolongation up to 510 ms (<460 ms), **(B)** ECG on admission to Cardiac Intensive Care Unit, right bundle branch hemiblock, **(C)** ECG after intravenous catecholamines, T-wave inversion, **(D)** ECG at 2 months follow-up, negative.

In our patient treated with chemotherapy and rituximab, TTS appeared after several courses of therapy. In the two cases of TTS associated with rituximab, the symptoms occurred within 48 hours (12) and within 40 minutes (13) after infusion of the monoclonal antibody, respectively. In this case where the patient was treated with chemotherapy and rituximab, the patient had suffered the death of a family member two months before the diagnosis of TTS and therefore, a doubt remains as to whether the patient was still undergoing psychological stress. Being over 60 years of age and having a poor performance status have a significant influence on the occurrence of thromboembolism in patients with newly diagnosed diffuse large B-cell lymphoma who have received rituximab plus vincristine, doxorubicin, cyclophosphamide (20). PE is considered as a potential trigger for secondary TTS (21), but TTS with PE is rarely reported (22). In the second case we used rituximab, plus vincristine, liposomal doxorubicin, cyclophosphamide. Doxorubicin and cyclophosphamide (13) together with rituximab have been associated with TTS. Doxorubicin alone has been related to TTS, too (23). In our second case the symptoms were more likely associated with TTS. In this patient, dobutamine and adrenaline were administered to reverse the worsening of the general condition. A surge in the circulating catecholamine level following sympathetic activation is suspected as a potential trigger of TTS (5) and there are case reports with TTS after receiving treatment with adrenergic agonist drugs, such as intravenous adrenaline (24), intramuscular adrenaline (25), local adrenaline (26), intravenous dobutamine (24, 27). A recent publication of pharmacovigilance confirmed the high correlation between adrenergic drugs and TTS (28). After a more careful sequential evaluation of cardiological examinations (**Figure 5**), it appears that TTS is related to intravenous catecholamines rather than to antiblastic therapy.

It seems there is a complex interconnection between cancer stressors, inflammation, and cytokine, which release excessive catecholamines. The mechanisms by which catecholamines can induce TTS seem different. In animal models, intracellular lipid droplets accumulate in cardiomyocytes in response to high doses of catecholamines as well as in endomyocardial biopsies of patients with TTS during the acute phase, but not after recovery (5). It is known that bevacizumab is associated with specific cardiovascular side-effects, in particular arterial thromboembolism (29). Izumiya et al. observed that a genetically engineered mouse model capable of blocking the VEGF signalling pathway showed increased dilatation of the ventricles and reduced contractile function, leading to heart failure (30). Yue Li et al. demonstrated that bevacizumab-mediated cardiotoxicity is associated with mitochondrial dysfunction and ERK pathway inactivation (31). Rituximab has also been shown to cause adverse cardiac events including arrhythmia and, on occasion, myocardial infarction. Kanamori et al. reported increased ventricular dysfunction after an infusion of rituximab, suggesting that transforming growth factor-β levels may have led to increased formation of reticulin fibre (diffusely present in cardiac muscles), causing a reduction in myocardial contractility and conduction (32). If we consider other monoclonal antibodies, Quagliarello et al. studied, for the first time, the putative cardiotoxic and pro-inflammatory effects of pembrolizumab associated to trastuzumab and they showed that such effects are mediated by overexpression of NF-kB and Leucotriene B4 related pathways (33). The main chemotherapeutic agents associated with TTS are: 5-fluorouracil, capecitabine, cytarabine, hydroxyurea, daunorubicin, cisplatin, docetaxel, paclitaxel (34). The occurrence of TTS during oncologic treatments is commonly attributed to direct cardiotoxicity of the treatment (mostly *via* free radicals-induced cardiac myocyte damage and death).

Therefore at a first interpretation of our two cases of TTS after bevacizumab, rituximab and chemotherapy we considered heart disease as an adverse drug reaction but a re-reading of the cases, after some time, allowed us to correlate TTS to causes other than the administration of monoclonal antibodies and chemotherapy. One limit of our study is its retrospective nature so we may have missed identifying some cases of TTS. Another limit lies in the difficulty in retrieving information on the potential triggers of TTS (e.g. emotional or painful stress triggering the cardiological episode).

One of the strong points of our study is that we considered a large time frame and a large number of onco-haematological patients that we could consider the low prevalence of TTS representative for cancer patients. Another strong point is that in our institute both oncologists and haematologists work side by side and share the same electronic medical records and therefore it was possible to search possible associations of TTS with different antineoplastic agents. One aspect that we can also highlight is that, to our knowledge, we are describing the first case of TTS from carcinosis subocclusion.

In conclusion, doctors often have difficulty differentiating between chemotherapy-induced cardiotoxicity and cardiac events unrelated to cancer treatment. Further research is warranted to understand whether bevacizumab, rituximab and chemotherapy can cause TTS. This is particularly important because these monoclonal antibodies and chemotherapy are widely used and their temporary or permanent suspension could compromise the success of treatment. In particular in our first case subocclusion seems to have been the most likely trigger of TTS rather than bevacizumab. In the second case, rituximab and/or chemotherapy could have been the initial trigger that led to embolism and subsequently to TTS but it is more likely that the infusion of catecholamines was the predominant trigger. Then it is important to investigate the real causes of TTS.

## Data Availability Statement

The raw data supporting the conclusions of this article will be made available by the authors, without undue reservation.

## Ethics Statement

Ethical review and approval was not required for the study on human participants in accordance with the local legislation and institutional requirements. The patients/participants provided their written informed consent to participate in this study.

## Author Contributions

MM designed the study. RV, PC, CG, and MF performed the literature search. MM, PC, CG, MF, LM, and GF collected and analysed the data. IB viewed the images of the CT scan. All authors contributed to data interpretation. MM drafted the manuscript. All authors contributed to the article and approved the submitted version.

## Conflict of Interest

The authors declare that the research was conducted in the absence of any commercial or financial relationships that could be construed as a potential conflict of interest.

## Publisher’s Note

All claims expressed in this article are solely those of the authors and do not necessarily represent those of their affiliated organizations, or those of the publisher, the editors and the reviewers. Any product that may be evaluated in this article, or claim that may be made by its manufacturer, is not guaranteed or endorsed by the publisher.

## Supplementary Material

Supplementary Video 1 Severe LV dysfunction with diffuse hypokinesis and basal sparing.
